# Epigenetic roles in the malignant transformation of gastric mucosal cells

**DOI:** 10.1007/s00018-016-2308-9

**Published:** 2016-07-27

**Authors:** Jun Tie, Xiangyuan Zhang, Daiming Fan

**Affiliations:** State Key Laboratory of Cancer Biology and Xijing Hospital of Digestive Diseases, Xijing Hospital, Fourth Military Medical University, No. 127, West Chang-Le Road, Xi’an, Shaanxi 710032 People’s Republic of China

**Keywords:** Epigenetic roles, Malignant transformation, Gastric mucosal cells, *Helicobacter pylori*

## Abstract

Gastric carcinogenesis occurs when gastric epithelial cells transition through the initial, immortal, premalignant, and malignant stages of transformation. Epigenetic regulations contribute to this multistep process. Due to the critical role of epigenetic modifications , these changes are highly likely to be of clinical use in the future as new biomarkers and therapeutic targets for the early detection and treatment of cancers. Here, we summarize the recent findings on how epigenetic modifications, including DNA methylation, histone modifications, and non-coding RNAs, regulate gastric carcinogenesis, and we discuss potential new strategies for the diagnosis and treatments of gastric cancer. The strategies may be helpful in the further understanding of epigenetic regulation in human diseases.

## Introduction

The latest figures from the World Health Organization (WHO) show that 951,600 new gastric cancer cases and 723,100 gastric cancer-related deaths occurred globally in 2012 [1]. The overall incidence of gastric cancer has declined. However, in China, the morbidity and mortality of gastric cancer rank 2nd and 3rd, respectively, among all malignant tumors [2]. Gastric cancer is classified into the following two subtypes: diffuse and intestinal. Usually, intestinal gastric cancers retain a glandular structure and undergo multiple processes as follows: chronic inflammation, atrophy, intestinal metaplasia, and atypical hyperplasia and eventually into gastric cancer. However, diffuse gastric cancer is relatively rare and is poorly differentiated to the extent that no glandular structure is recognizable. At present, no clear precancerous lesions of diffuse gastric cancer have been defined [3, 4]. *Helicobacter pylori* (*H. pylori*) infection plays an important carcinogenic role in both subtypes of gastric cancer. Many trials have demonstrated the possibility of cancer prevention through *H. pylori* screening and eradication. The malignant transformation of gastric mucosa involves multimolecular events, including gene mutation [5] and epigenetic alteration [6]. This study presents a review of the roles of epigenetic alterations in the malignant transformation of gastric mucosa.

## The concept and significance of epigenetics

The concept of epigenetics was first proposed by Waddington [7]. Epigenetics refers to the heritable changes in gene expression that are independent of variations in DNA sequences. The main types of epigenetic processes include DNA methylation, histone modification, and chromatin remodeling as well as the function of non-coding RNA (ncRNA). The basic theory of classical genetics cannot adequately explain the biodiversity within species. For example, identical twins carrying the same DNA sequences may exhibit distinct phenotypes and different susceptibility to diseases. The proposal of epigenetics has compensated for such shortcoming of classical genetic theory. Epigenetics is a component of normal physiological regulation, and abnormal epigenetic regulation may lead to tumorigenesis. Studies have suggested that intestinal-type gastric cancer originates from chronic gastritis, which gradually progresses through stages of chronic atrophic gastritis, intestinal metaplasia, and atypical hyperplasia and ultimately develops into advanced gastric cancer [8, 9]. During the malignant transformation of gastric mucosa, a large number of genes are subjected to epigenetic regulation. The genes show cumulative changes as the disease evolves [10, 11].

## Methylation of tumor suppressor genes is an important mechanism responsible for malignant transformation of gastric mucosa

Methylation is a type of chemical modification that occurs in DNA sequences. In mammalian cells, DNA methylation occurs almost exclusively at the fifth carbon atom of the cytosine residues within cytosine–phosphate–guanine (CpG) dinucleotides. CpG dinucleotides tend to form CG-rich clusters called CpG islands. CpG islands are mainly distributed in the core promoter sequence and transcription start site of structural genes. DNA methylation may induce changes in chromatin structure, DNA conformation, DNA stability, and the interactions between DNA and protein, resulting in transcription inhibition [12]. Two adverse phenomena characterize the process of carcinogenesis: locus-specific hypermethylation and global depletion of methyl groups from cancer genomes. Hypermethylation of promoters has been widely shown to contribute to the silencing of tumor suppressor genes during carcinogenesis. Global hypomethylation of the cancer genome was initially shown to cause genome-wide allelic instability, but recently, the involvement of this process in transcriptional gene regulation has become increasingly recognized [13, 14].

Promoter hypermethylation-induced inactivation of tumor suppressor genes is an important mechanism that leads to gastric carcinogenesis [15]. For example, CDH1, the gene encoding epithelial cadherin (E-cadherin), is a tumor suppressor gene located on chromosome 16q22.1. E-cadherin is expressed in normal epithelium and plays a role in calcium-dependent cell adhesion. CDH1 is hypermethylated in 40–80 % of human primary gastric carcinoma. In diffuse gastric cancer, a methylation-induced decrease in E-cadherin expression has been observed in more than 50 % of the undifferentiated early cancers and adjacent non-cancerous gastric epithelial tissues. Therefore, CDH1 methylation-induced loss of E-cadherin expression is an early event in the malignant transformation of gastric mucosa [16, 17]. E-cadherin is also inactivated by mutation and accounts for the hereditary nature of diffuse-type gastric cancer [18]. Runt-related transcription factor 3 (RUNX3) is a key molecule in the transforming growth factor-β (TGF-β) signaling pathway. The expression of RUNX3 is significantly reduced in gastric cancer. The main reason for the decreased RUNX3 expression is DNA hypermethylation in the promoter region. Kim et al. found that RUNX3 CpG island methylation occured in 8.1 % of chronic gastritis cases, 28.1 % of intestinal metaplasia cases, 27.3 % of gastric adenocarcinoma cases, 64 % of primary gastric cancer cases, and 60 % of gastric cancer cell lines [19]. In RUNX3 knockout mice, apoptosis is inhibited. These mice show hypertrophy of gastric mucosa and intestinal metaplasia of gastric epithelial cells, indicating that RUNX3 hypermethylation plays an important role in the malignant transformation of intestinal-type gastric cancer [20, 21]. In addition, chronic gastritis, intestinal metaplasia, gastric adenoma, and gastric cancer show an increasing frequency of p16/cyclin-dependent kinase inhibitor 2A (CDKN2A) methylation [22–24]. This finding indicates that p16/CDKN2A methylation occurs at the initial stage of gastric mucosal malignant transformation and undergoes cumulative change as the disease progresses. Genes related to the malignant transformation of gastric mucosa that undergoes promoter methylation also include the retinoblastoma (RB) gene, von Hippel–Lindau (VHL) tumor suppressor gene, breast cancer 1 (BRCA1) gene, human mutL homolog 1 (hMLH1) gene, X-ray repair cross-complementation group 1 (XRCC1) gene, and ADAM metallopeptidase with thrombospondin type 1 motif 9 (ADAMTS9) gene [25, 26].

In gastric cancer, the distribution characteristics of gene methylation are correlated with biological tumor characteristics and patient prognosis. Patrick Tan and colleagues investigated DNA methylation profiles of 240 primary gastric cancers and gastric cancer cell lines [27]. It has been found that methylomes are widely distributed in gastric cancers. However, these results need to be further verified. In addition, previous data on the methylation of gastric mucosal transformation-related genes are mainly derived from experimental studies of previously established cell lines or small-sized clinical tissue specimen studies. The current knowledge on gene methylation is far from being accurate and comprehensive. Future studies focusing on the following aspects will be more valuable: (1) longitudinal studies: large-scale DNA methylation profiling of clinical tissue specimens—longitudinal, dynamic cohort studies that analyze serial clinical specimens obtained from individual patients at various stages, from inflammation, intestinal metaplasia, and atypical hyperplasia to gastric cancer, are particularly inadequate; a genome-wide longitudinal study of DNA methylation based on such specimens will provide more accurate and comprehensive results; (2) data mining: collection and analysis of the data on gene methylation in normal gastric mucosa, precancerous lesions, and gastric cancer in various populations—exploration of the methylation pattern changes that occur during the malignant transformation of gastric mucosa using large-scale data mining allows a complete understanding of the gene methylation characteristics related to the malignant transformation of gastric mucosa as well as the differentiation of the key methylation changes from the numerous accompanying changes; and (3) non-CpG methylation: non-CpG methylation is an emerging field of research [28, 29]. However, a few data have been obtained for gastric cancer. The research on the distribution, recognition, and regulation of non-CpG methylation in gastric cancer will further deepen our understanding of epigenetic regulation in the transformation of gastric mucosa.

## The histone code affects the malignant transformation of gastric mucosa

In eukaryotes, DNA, histones, and non-histone proteins are arranged in a highly ordered pattern to form chromatin. Histones are divided into five classes, including nucleosomal core histones (H2A, H2B, H3, and H4) and linker histones (H1). Each core histone consists of a globular structural domain and an N-terminal tail that is exposed on the surface of the nucleosome. A variety of covalent modifications may occur at the N-terminus of the core histones, including acetylation, methylation, phosphorylation, ubiquitination, and glycosylation. These histone modifications alter the chromatin structure and, therefore, determine the state of gene activation/inactivation; these modifications also regulate physiological processes in the cells. Different histone modifications are orchestrated in both time and space, forming a complex regulatory network known as the “histone code.” Numerous studies have demonstrated histone modification changes in gastric cancer, and such changes are of great clinical significance [30, 31]. Recent studies of gastric cancer-related histone modifications have mainly focused on histone acetylation, methylation, and phosphorylation.

Histone acetylation: histone acetylation is coregulated by histone acetyltransferases (HATs) and histone deacetylases (HDACs). Histone acetylation promotes transcription, whereas histone deacetylation inhibits transcription. Abnormal expression of HDACs and HATs is frequently observed in gastric precancerous lesions and gastric cancer [32, 33]. Current studies have found that histone deacetylation occurs at the promoter region of a number of genes in gastric cancer, including p21(WAF1/CIP1) [34], RIP-associated ICH1/CED3-homologous protein with a death domain (RAIDD) [35], DTW domain containing 1 (DTWD1) [36], p53 upregulated modulator of apoptosis (PUMA) [31], gelsolin and retinoic acid receptor beta [37], deleted in liver cancer-1 (DLC1) [38], and thioredoxin-interacting protein (TXNIP) [39]. In addition, histone deacetylation has been shown to be positively correlated with the downregulated expression of the above genes.

Histone methylation: histone methylation mainly occurs at the lysine (K) and arginine (R) residues of H3 and H4 and is regulated by histone methyltransferases (HMTs) and histone demethylases (HDMs). There are three types of histone methylation: monomethylation, dimethylation, and trimethylation. Different sites and types of histone methylation confer different functions. Methylation of H3K9 and H4K20 inhibits gene expression, whereas methylation of H3K4, H3K36, and H3K79 activates gene expression. H3K27 monomethylation activates gene expression, whereas H3K27 dimethylation and trimethylation inhibit gene expression. For example, H3K27 trimethylation inhibits the expression of Arg kinase-binding protein 2 (ArgBP2) in gastric cancer [40].

Histone phosphorylation: phosphorylation can disrupt the interaction between histones and DNA, and renders the chromatin structure unstable. In addition, phosphorylation may create a surface that binds to protein recognition modules, thereby allowing interaction with specific protein complexes. These two mechanisms enable histone phosphorylation to play a role in chromosome condensation/separation, transcription activation, apoptosis, and DNA damage repair. Studies on the role of histone phosphorylation in the malignant transformation of gastric mucosa are insufficient. Fehri et al. found that *H. pylori* infection reduces the phosphorylation levels of histone H3S10 and H3T3 in gastric epithelial cells, thus regulating the cell cycle [41]. This finding may represent an important mechanism of *H. pylori*-induced gastric carcinogenesis. In addition, an increased phosphorylation level of histone H3 was shown to be closely related to the histological type, vascular infiltration, and lymph node metastasis of gastric cancer and is an independent factor associated with a poor prognosis in patients with gastric cancer [42]. The findings indirectly support the hypothesis that histone phosphorylation is involved in the malignant transformation of gastric mucosal cells.

However, relevant studies remain focused on the relationships between the changes in the overall level of certain histone modifications and various pathological states during gastric mucosal carcinogenesis. Studies investigating the mechanisms through which histone modifications affect the malignant transformation of gastric mucosa are currently lacking. To pinpoint the genes or signaling pathways through which histone modifications affect the malignant transformation of gastric mucosa, studies that utilize histone modification-specific antibodies to coprecipitate chromatin or those that employ oligonucleotide microarrays or deep DNA sequencing to identify significantly differentially modified gene loci/chromatin segments and then combine these discoveries with gene function verification would be beneficial.

## Non-coding RNAs regulate the malignant transformation of gastric mucosal epithelial cells

Non-coding RNA (ncRNA) is a general term for an RNA molecule that does not encode a protein. ncRNAs include micro RNA (miRNA), piwi-interacting RNA (piRNA), long non-coding RNA (lncRNA), transfer RNA (tRNA), and ribosomal RNA (rRNA). A large number of studies have focused on the roles of miRNAs and lncRNAs in gastric carcinogenesis.

### MiRNAs

MiRNAs are a class of evolutionarily conserved, endogenous, non-protein-coding small RNAs. MiRNAs participate in the malignant transformation of gastric mucosal cells by negatively regulating the expression of target genes.

#### Abnormal expression of miRNA molecules in gastric cancer

Petrocca et al. compared the miRNA expression profiles between tissues with histological signs of chronic gastritis and normal gastric mucosa [43]. It has been found that, in chronic gastritis, the expression of miR-1 and miR-155 is upregulated, whereas the expression of miR-20, miR-26b, miR-202, miR-203, and miR-205 is downregulated. Ueda et al. examined miRNA expression in 160 paired samples of gastric cancer tissues and non-cancerous tissues [44]. The authors found that the expression of 22 miRNAs is upregulated, while the expression of 13 miRNAs is downregulated in gastric cancer tissues compared with the non-cancerous tissues. In addition, 83 % of the patients with gastric cancer could be accurately diagnosed based on miRNA expression profiles in tissue specimens. Microarray analysis has been performed to examine miRNA expression in gastric cancer tissue specimens collected in a large number of countries and geographical regions. The results have shown that the expression of miR-21 [45], miR-27a [46] and miR-196a [47] is significantly elevated in gastric cancer, whereas the expression of lethal-7 (let-7) miRNA [48], miR-101 [49, 50], and miR-29a [51] is markedly reduced. These results clearly demonstrate that miRNAs are involved in gastric carcinogenesis. However, the consistency of previous results is poor. This may be due to sample variation. At present, there is no generally accepted characteristic miRNA expression profile of gastric cancer. Further studies of large and multicenter sample cohort(s) are needed.

#### Abnormal expression of miRNAs significantly affects the malignant phenotype of gastric cancer cells

**Fig. 1:**
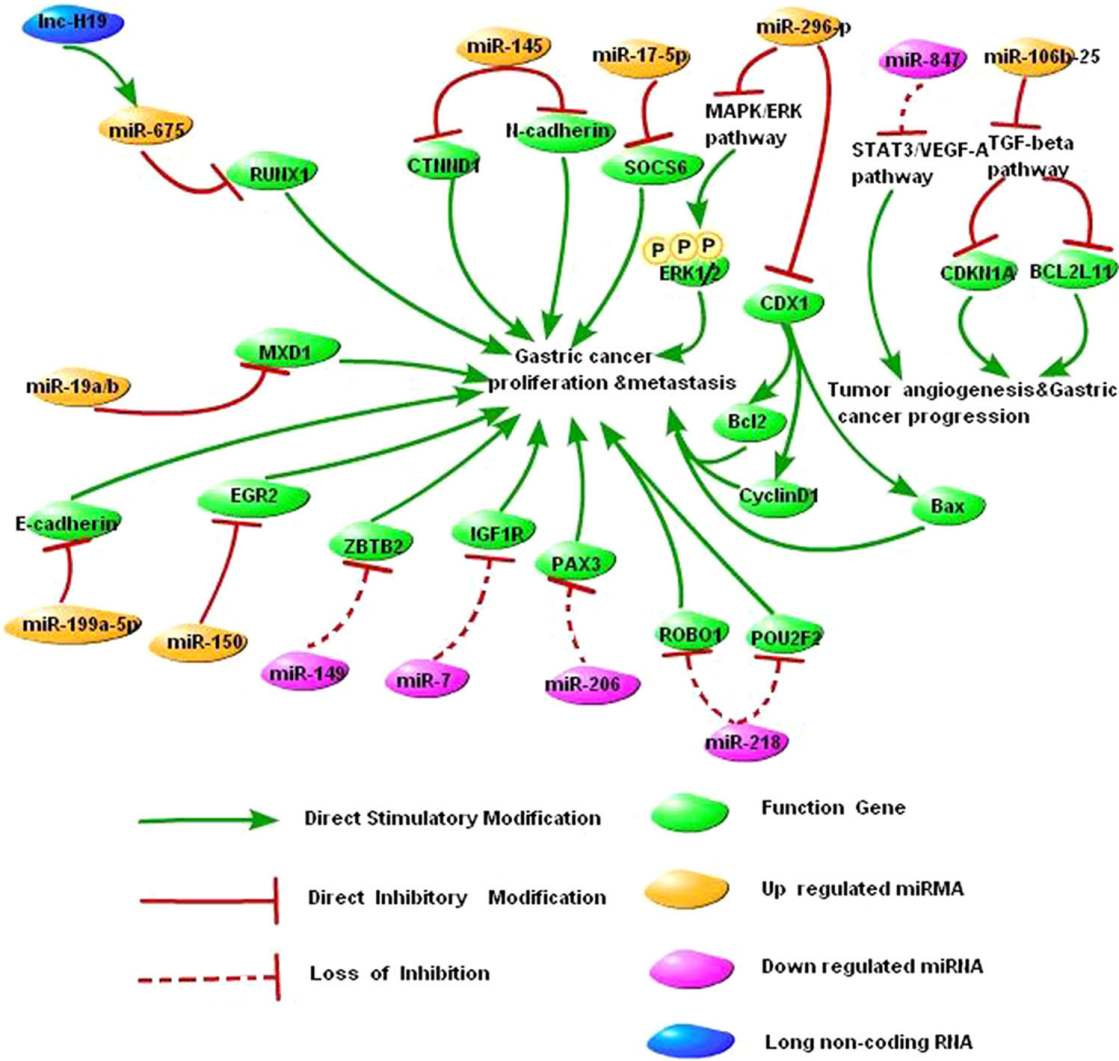
NcRNA-oriented network in the malignant transformation process of gastric cancer. This figure provides insight into the roles of ncRNA and its related protein in the malignant transformation of gastric mucosal cells. It can be seen that a complex network composed of ncRNA and its upstream and downstream components affects the malignant transformation of gastric mucosal cells

The expression of miR-847 is decreased in gastric cancer, which activates the signal transducer and activator of transcription 3 (STAT3)/vascular endothelial growth factor A (VEGF-A) pathway, increases tumor angiogenesis, and promotes the development and progression of gastric cancer [52]. The expression of miR-145 is upregulated in gastric cancer, which inhibits the expression of catenin (cadherin-associated protein), delta 1 (CTNND1), and N-cadherin while promoting the translocation of CTNND1 and E-cadherin from the cytoplasm to the cell membrane. As a result, the proliferation and metastasis of gastric cancer cells are promoted, and the apoptosis of gastric cancer cells is inhibited [53]. The expression of the miR-106b-25 cluster is upregulated in gastric cancer, which inhibits the TGF-β pathway, induces the downregulation of the expression of cyclin-dependent kinase inhibitor 1A (CDKN1A) and BCL2-like 11 apoptosis facilitator (BCL2L11), and promotes the development and progression of gastric cancer [43]. Our study demonstrated that the expression of miR-17-5p is significantly increased in gastric cancer tissues. High miR-17-5p expression inhibits suppressor of cytokine signaling 6 (SOCS6), which promotes the proliferation of gastric cancer cells [54]. The expression of miR-296-5p is abnormally increased in gastric cancer, which inhibits the expression of caudal-related homeobox 1 (CDX1). Furthermore, miR-296-5p/CDX1 affects the phosphorylation level of the extracellular signal-regulated kinases 1 and 2 (ERK1/2) through the mitogen-activated protein kinase (MAPK)/ERK pathway and induces changes in the expression levels of the cell cycle-related protein cyclin D1 and the apoptosis-related proteins B-cell lymphoma 2 (Bcl2) and BCL2-associated X (Bax), thus maintaining the survival of gastric cancer cells and regulating cell proliferation [55]. In addition, miR-150 [56], miR-149 [57], miR-7 [58], miR-199a-5p [59], miR-206 [60, 61], miR-19a/b [62], and miR-218 [63, 64] are all involved in the development and progression of gastric cancer (Fig. 1).

### LncRNA

LncRNA refers to a class of RNA molecules greater than 200 nucleotides (nt) in length that are transcribed mainly by RNA polymerase II, that lack apparent open reading frames (ORFs) and that do not encode proteins. However, lncRNAs participate in the regulation of a variety of intracellular signaling processes (including tumorigenesis) through the modification of chromatins, activation of transcription, and interference with transcription. Recent studies have shown that several lncRNAs are abnormally expressed in gastric cancer. Moreover, abnormal expression of lncRNAs plays an important role in the development, progression, invasion, and metastasis of gastric cancer.

HOX transcript antisense intergenic RNA (HOTAIR) is located on chromosome 12q13.13. HOTAIR is a 2158-nt lncRNA that possesses a trans-regulatory function. The 5′ end of HOTAIR binds to the initiation complex known as polycomb repressive complex 2 (PRC2). Binding of HOTAIR to PRC2 induces the phosphorylation of EZH2, a subunit of PRC2, at threonine 345 and the subsequent trimethylation of chromosome-bound histone H3K27, thereby inhibiting the expression of the target genes. In addition, the 3′ end of HOTAIR binds to the lysine-specific demethylase 1 (LSD1)/REST corepressor 1 (CoREST)/repressor element 1 (RE1) silencing transcription factor (REST) complex, which mediates histone H3K4me2 demethylation and, thereby, regulates the transcriptional activity of target genes. HOTAIR expression is significantly increased in gastric cancer tissues compared with paracancerous tissues. In diffuse gastric cancer, the high HOTAIR expression group exhibits drastically increased invasion and lymph node metastasis and a decreased overall survival rate in comparison with the low HOTAIR expression group. In addition, studies have shown that inhibition of HOTAIR expression in gastric cancer cells decreases the expression of matrix metalloproteinases 1 and 3, reduces the invasive capability of cancer cells, and reverses the epithelial–mesenchymal transition (EMT) in gastric cancer cells. These findings demonstrate that HOTAIR plays an important role in the development and progression of gastric cancer [65–67]. The H19 gene (full length: 2.5 kb) is located on the human chromosome 11p15.5 region and contains a total of 5 exons and 4 introns. The processed, mature H19 has a length of 2.3 kb. Due to the lack of obvious ORFs, H19 is defined as an lncRNA. H19 expression is significantly elevated in gastric cancer tissues compared with paracancerous tissues [68]. Overexpression of H19 enhances the proliferative capacity of the cells, whereas small interfering RNA (siRNA)-mediated interference of H19 expression enhances apoptosis. The effects of overexpression and downregulation of H19 are related to the inactivation and activation of the TP53 gene. Recent studies have shown that transcription of the H19 gene also produces a mature miRNA, namely, miR-675. H19 is capable of regulating the progression of gastric cancer through the H19/miR-675/runt-related transcription factor 1 (RUNX1) signaling axis [69, 70]. In addition, tumor suppressor candidate 7 (TUSC7) [71], maternally expressed 3 (MEG3) [72], BM742401 [73], colon cancer-associated transcript 1 (CCAT1) [74], and multidrug resistant (MDR)-related and upregulated lncRNA (MRUL) [75] have been found to be differentially expressed between gastric cancer cells and normal gastric mucosal cells and affect the malignant phenotype of gastric cancer cells.

Current studies in the field of ncRNAs have mainly focused on the effects of such molecules on the malignant phenotypes of gastric cancer cells, including growth, proliferation, metastasis, and drug resistance. The conclusion that ncRNAs participate in the malignant transformation of gastric mucosal cells is based on the findings that ncRNAs are differentially expressed between normal gastric mucosal cells and gastric cancer cells and that the differential expression of ncRNAs induces functional changes in certain malignant phenotype of gastric cancer cells. There are virtually no functional studies that directly address the malignant transformation of normal gastric mucosa. In addition, the intrinsic link between various ncRNA molecules remains unclear. We simulated the interactions between a number of gastric carcinogenesis-related molecules that have been identified by our study or reported in the literature. However, further biological experiments are required to discover the ncRNA-regulated network.

## *H. pylori* infection promotes gastric cancer mainly through epigenetic regulation

*H. pylori* infection is the most important risk factor for gastric cancer. The epigenetic changes induced by *H. pylori* compose one of the principal molecular mechanisms of gastric carcinogenesis.

### *H. pylori* infection and gene methylation

Numerous studies have demonstrated that *H. pylori* infection is closely related to abnormal CpG island methylation. Maekita et al. found that methylation levels of all the detected regions were much higher in *H. pylori*-positive samples than in *H. pylori*-negative samples among healthy volunteers [76]. Nakajima et al. analyzed the promoter methylation of CpG islands of 48 genes that may be methylated in gastric cancer cell lines. The results showed that 26 genes were consistently methylated in individuals with current or past infection by *H. pylori* [77]. Shin et al. identified quite distinct methylation profiles according to the presence or absence of current *H. pylori* infection in non-cancerous gastric mucosae from patients with gastric cancer [78]. Cheng concluded that FOXD3-mediated transcriptional control of tumor suppressors is deregulated by *H. pylori* infection-induced hypermethylation. This in turn could affect the suppression of gastric tumors [79]. These findings indicate that *H. pylori* infection potently induces CpG island methylation and may be responsible for the initiation of gastric carcinogenesis.

*H. pylori*-mediated chronic inflammation is one of the important causes of DNA methylation. A number of studies have suggested that methylation levels in the gastric mucosa after *H. pylori* infection decrease after *H. pylori* eradication [80, 81]. These data support the idea that *H. pylori*-mediated inflammation induces methylation. However, how the inflammation triggers DNA methylation is not yet known.

### *H. pylori* infection and histone modification

A relatively few studies have investigated whether *H. pylori* affects histone modifications. The infection of gastric epithelial cells with *H. pylori* leads to hyperacetylation of histone H4 [82], which induces the binding of histone H1 to ATP [83] and causes histone H3 dephosphorylation and deacetylation [41, 84]. These changes result in the abnormal expression of oncogenes and tumor suppressor genes [85, 86], which contributes to malignant transformation of gastric epithelial cells.

### *H. pylori* infection is a major cause of abnormal miRNA expression

MiRNAs play an important role in *H. pylori* infection-induced malignant transformation of gastric mucosa. Zhang et al. demonstrated for the first time that *H. pylori* infection is able to induce changes in miRNA expression profiles [87]. They found that miR-21 expression is significantly increased in *H. pylori*-positive gastric tissues, indicating that the increased expression of miR-21 may be related to *H. pylori* infection. In AGS human gastric carcinoma cells, *H. pylori* infection promotes the secretion of nuclear factor kappa B (NF-κB) and interleukin 6 (IL-6) and activates activator protein 1 (AP-1) and STAT3, resulting in significantly upregulated miR-21 expression and drastically enhanced cell proliferative and invasive capabilities. Using miRNA microarrays, Matsushima et al. identified 55 miRNAs that were differentially expressed between *H. pylori*-positive and *H. pylori*-negative endoscopic biopsy specimens [88]. Among the 55 miRNAs, the expression of 30 miRNAs was significantly reduced. A portion of the miRNAs (including miR-223, miR-375, and miR-200c) was found to be significantly correlated with gastric mucosal inflammatory activity, chronic inflammation, and *H. pylori* infection severity scores. Correlation analysis showed that 8 miRNAs can be used to accurately predict whether *H. pylori* infection is present. Infection of the cells with an *H. pylori* strain containing the wild-type CagA (cytotoxin-associated gene A) structural domain induced changes in the expression of certain miRNAs (e.g., let-7, miR-125a, and miR-500), whereas *H. pylori* strains with mutant CagA showed no such effect. Studies conducted by Saito et al. showed that miR-17 and miR-20a are also involved in the gastric cancer-promoting signaling pathways mediated by CagA [89, 90]. CagA activates c-Myc through the activation of the Erk pathway, which further stimulates the expression of miR-17 and miR-20a. MiR-20a is capable of suppressing p21 expression. In addition, miR-146a [91], miR-155 [92], and miR-218 [93] are also involved in *H. pylori* infection-related malignant transformation of gastric mucosa. In a study conducted by Matsushima et al., patients who tested positive for *H. pylori* infection were successfully cured with an anti-*H. pylori* regimen and were reexamined 4 weeks after eradication of *H. pylori* infection [88]. Cure of the *H. pylori* infection not only restored the levels of 14 miRNAs whose expression was downregulated during *H. pylori* infection but also significantly reduced the levels of a portion of the miRNAs whose expression was upregulated by *H. pylori* infection. This phenomenon indicates that downregulation/inhibition of the expression of cancer-promoting miRNAs using methods, such as oligonucleotides and miRNA sponges combined with the introduction of exogenous cancer-suppressing miRNAs, may reduce or even partially block the promoting effect of *H. pylori* on gastric cancer.

**Fig. 2:**
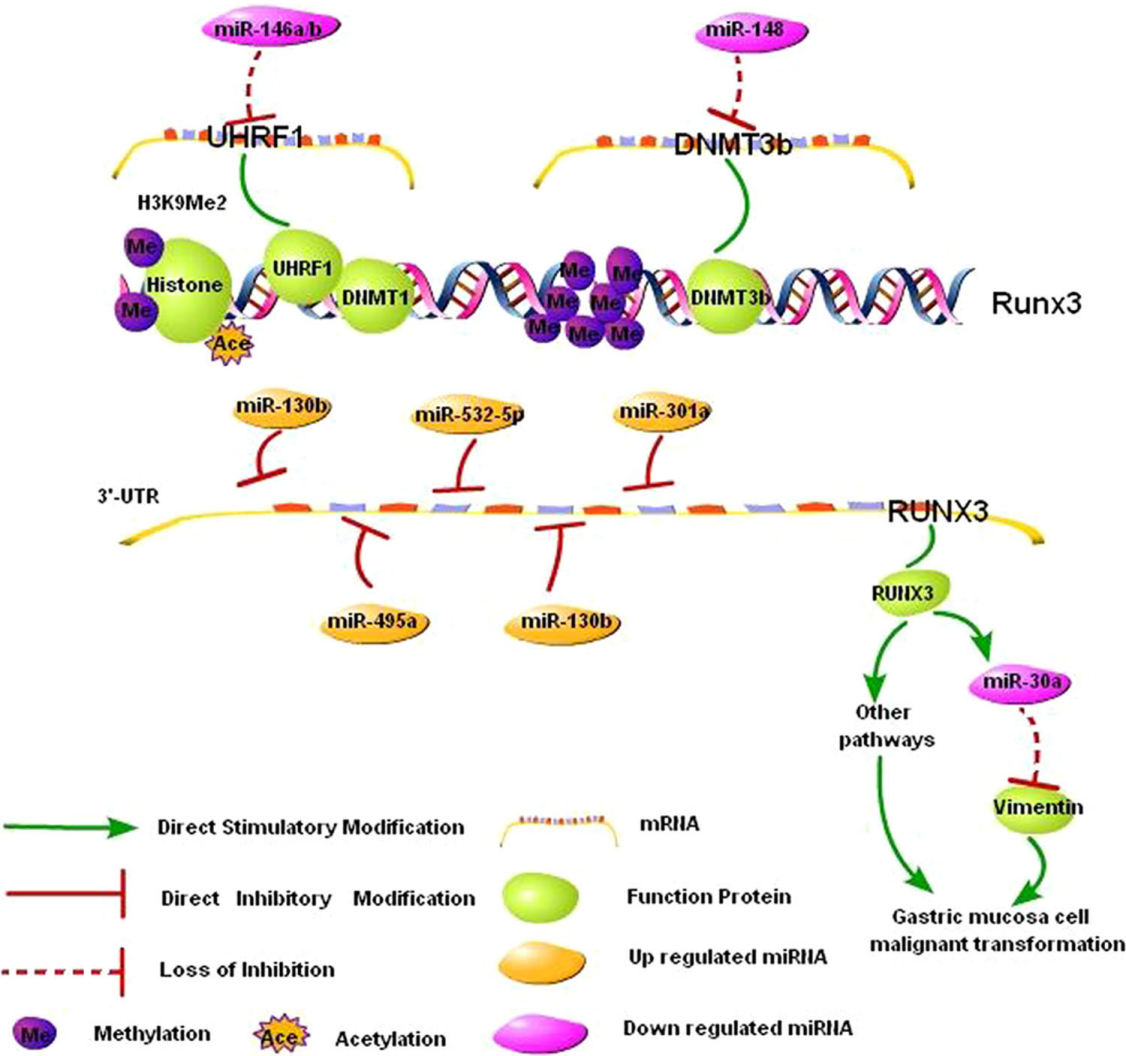
Epigenetic regulation of RUNX3 in the malignant transformation of gastric mucosal cells. MiR-146a/b and miR-148 directly inhibited UHRF1 and DNMT3b, respectively. Downregulation of miR-146a/b and miR-148 led to the increase in UHRF1 and DNMT3b, and this effect in turn inactivated RUNX3 via promoter methylation in gastric cancer. In addition, increased H3K9 dimethylation and reduced H3 acetylation, as well as the increased miR-130b, miR-301a, miR-106a, miR-103a, miR-495, and miR-532-5p, synergistically inhibited the expression of RUNX3

The effects of various types of epigenetic regulations (such as DNA methylation, histone modification, and ncRNA) on the malignant transformation of gastric mucosa are not independent. Instead, the epigenetic effects interact synergistically to promote gastric carcinogenesis. In addition, different epigenetic changes may coordinate in the regulation of the expression of one carcinogenesis-related gene (Fig. 2). For example, ubiquitin-like containing PHD and RING finger domains 1 (UHRF1) is known to maintain DNA methylation via the recruitment of DNA methyltransferase 1 (DNMT1) [94]. We identified and verified miR-146a/b as direct upstream regulators of UHRF1 [95]. Duursma et al. found that miR-148 targets human DNMT3b [96]. MiR-146a/b and miR-148 can regulate RUNX3 expression via the effects of UHRF1 and DNMT1 on promoter methylation. In addition, increased H3K9 dimethylation and reduced H3 acetylation synergistically inhibit the transcription of RUNX3 [97, 98]. Moreover, miR-130b [99], miR-301a [100], miR-106a [101], miR-103a [102], miR-495 [103], and miR-532-5p [104] directly inhibit RUNX3 translation at the post-transcriptional level. Decreased RUNX3 expression directly downregulates miR-30a expression, which enhances the expression of the miR-30a target gene vimentin and promotes EMT in gastric cancer cells [105]. RUNX3 regulates gastric cancer cell proliferation via the TGF-β [106] and Wnt [107, 108] pathways and affects angiogenesis in gastric cancer by regulating the expression of vascular endothelial growth factor (VEGF) [109]. In contrast, different epigenetic changes may regulate the expression of different carcinogenesis-related genes, whereby they cooperate to promote the malignant transformation of gastric mucosa.

## Conclusions

The exploitation of characteristic epigenetic alterations during the malignant transformation of gastric mucosa allows for the prevention, diagnosis, treatment, and prognostic evaluation of gastric cancer from a new perspective independent of protein expression. For example, it has been reported that promoter methylation of death-associated protein kinase (DAPK) [110], E-cadherin [111], and p16 [112] genes may serve as a criterion for sensitive and specific diagnosis of gastric cancer. We have found that the methylation status of the ring finger protein 180 (RNF180) gene may be used to predict the malignant potential of intestinal metaplasia and atypical hyperplasia of gastric mucosa and diagnose early gastric cancer (unpublished data). Currently, we have established a fluorescence-based quantitative technique that enables the analysis of RNF180 gene methylation and meets the registration requirements for diagnostic reagents. We have initiated a clinical trial application to test this diagnostic kit.

The reversibility of epigenetic alterations (such as DNA methylation and histone modification) has recently become a hot topic in drug development. Studies have found that inhibition of deacetylase may suppress the malignant phenotype of gastric cancer cells [39, 113–116] and increase the sensitivity of gastric cancer cells to chemotherapy [115, 117, 118]. Histone deacetylase inhibitors, such as Vorinostat (Zolinza, suberoylanilide hydroxamic acid, SAHA) developed by Merck & Co., Inc. (USA) and Chidamide developed in China, have been successfully used in the clinical treatment of cancer [119–121]. Currently, a number of DNA methyltransferase inhibitors (DNMTi) and histone deacetylase inhibitors are undergoing clinical trials to assess their safety and efficacy for the treatment of tumors [122, 123].

However, unfavorable epigenetic changes induced by epigenetic drugs may lead to severe side effects. The discovery of ways by which the specificity against tumor cells can be enhanced and the side effects can be reduced is still a hot topic. The epigenetic modifications and their combinations that are involved in the malignant transformation of gastric mucosa are highly complex and diverse. There are many outstanding issues requiring clarification. In-depth studies and further elucidation of the epigenetic networks that regulate the malignant transformation of gastric mucosa will provide a wealth of pathways and targets for understanding gastric cancer development and progression, conducting molecular typing, establishing new therapies, and developing new drugs.
